# Obesity Does Not Influence Outcomes in Hepatocellular Carcinoma Patients following Curative Hepatectomy

**DOI:** 10.1371/journal.pone.0125649

**Published:** 2015-05-12

**Authors:** Zhe Guo, Jun Zhang, Jing-Hang Jiang, Le-Qun Li, Bang-De Xiang

**Affiliations:** 1 Department of Hepatobiliary Surgery, Affiliated Tumor Hospital of Guangxi Medical University, Nanning, P.R. China; 2 Department of Thyroid and Breast Surgery, The Central Hospital of Wuhan, Wuhan, P.R. China; 3 Department of Ultrasound, Wuhan NO. 1 Hospital, Wuhan, P.R. China; 4 Department of General Surgery, The Second People’s Hospital of Jing Men, Jingmen, P.R. China; The Chinese University of Hong Kong, HONG KONG

## Abstract

**Background:**

Whether obesity affects surgical outcomes in patients with hepatocellular carcinoma (HCC) is controversial. Here we retrospectively evaluated the impact of obesity on outcomes in HCC patients after curative hepatectomy.

**Methods:**

Patients with Child-Pugh A liver function who underwent curative hepatectomy between 2006 and 2010 were categorized as obese (BMI ≥25 kg/m^2^, n = 68) and non-obese (<25 kg/m^2^, n = 242). To reduce interference from baseline differences between the two groups, propensity score-matched analysis was performed in the ratio 1:2 using a caliper width of 0.1. Surgical outcomes were compared for 61 obese and 115 non-obese patients.

**Results:**

Obese patients had higher levels of albumin and aspartate aminotransferase, and more solitary tumors compared to the non-obese patients (all *P*<0.05). In the propensity-matched cohort, baseline characteristics did not differ between the two groups (all *P*>0.05). Obese and non-obese patients had comparable 30-day mortality (1.6% vs. 2.6%, *P* = 1.000), 90-day mortality (3.3% vs. 4.3%, *P* = 1.000), and incidence of postoperative complications (19.7% vs. 18.3%, *P* = 0.819). Overall survival at 1, 3, and 5 years was similar for obese patients (83.6%, 63.6%, 41.6%) as for non-obese patients (80.9%, 65.9%, 49.1%; *P* = 0.358). Disease-free survival at 1, 3, and 5 years was also similar for obese patients (71.5%, 36.3%, 24.3%) as for non-obese ones (60.2%, 43.7%, 27.7%; *P* = 0.969).

**Conclusion:**

Our propensity score-matched analysis strengthens the case that obesity does not adversely affect surgical outcomes of HCC patients undergoing curative hepatectomy.

## Introduction

Hepatocellular carcinoma (HCC) is the fifth most common malignancy in the world, and its incidence and mortality rate are increasing [1]. Surgical resection remains the most effective and practical treatment for HCC patients [2]. As surgical techniques as well as peri- and postoperative management of HCC patients have improved, morbidity and mortality rates have fallen significantly [3]. Nevertheless, long-term survival remains unsatisfactory owing to the high rates of recurrence and metastasis [4]. These poor clinical outcomes highlight the need for better understanding of the factors that affect prognosis. Such insights may improve decision-making about therapeutic modalities to treat HCC and reduce the risk of recurrence and metastasis.

Obesity is a serious global health problem: approximately 1.5 billion people around the world are overweight, and 671 million are obese [5]. Obesity, already linked to several disorders collectively known as "metabolic syndrome", including hypertension, cardiovascular disease and diabetes mellitus, is also now widely accepted as a significant risk factor for the development of various malignancies, including HCC [6,7].

Studies investigating whether obesity affects post-resection survival of HCC patients have given conflicting results. Some authors have reported lower overall survival (OS) or disease-free survival (DFS) rate in obese patients than in non-obese ones [8–10], while others have reported no significant differences between the two types of patients [11–17]. To help resolve the question of whether obesity influences postoperative outcomes in HCC patients, we retrospectively evaluated the influence of obesity on rates of postoperative complications, OS, and DFS in HCC patients.

## Patients and Methods

The study protocol was approved by the institutional review board of the Tumor Hospital of Guangxi Medical University, Nanning, China. Written informed consent had been obtained from all patients prior to undergoing resection.

### Patients

310 consecutive HCC patients with Child-Pugh A liver function who underwent curative hepatectomy as initial treatment at our hospital between December 2006 and December 2010 were eligible for inclusion in this study. BMI was calculated for each patient before surgery, and patients were classified into an obese group (BMI ≥25 kg/m^2^) or a non-obese group (BMI <25 kg/m^2^). World Health Organization (WHO) defines a BMI above 30 kg/m^2^ for obesity. However, in China, the prevalence of individuals with such a BMI is no more than 3% [18], in contrast to 20%-30% prevalence in Western countries [19]. In China, the definition of obesity is proposed to be a BMI ≥25 kg/m^2^ because of the incidence of obesity-related disorders increases with a BMI ≥25 kg/m^2^ [20].

### Curative hepatectomy

Indications for surgery were as follows: lack of ascites, hypersplenism, appropriate residual liver determined by volumetric computed tomography (CT), and presence of Child-Pugh A liver function [21]. Curative hepatectomy was defined to involve (1) complete removal of all nodules with the resection margin greater than 10 mm, (2) the absence of invasion of the main trunk and first-order branches of the portal vein, common hepatic duct and its first-order branches or main trunk of the hepatic vein and inferior vena cava (3) the absence of intra- or extra-hepatic metastasis, and (4) the absence of residual tumor or portal tumor thromboses on postoperative imaging. HCC diagnosis was confirmed by histopathological examination of surgical samples. Major resection was defined as the resection of three or more segments according to Couinaud’s classification [22,23]. The hepatectomy technique was performed as described [24].

### Definitions of postoperative complications

Liver failure was defined based on increased international normalized ratio and hyperbilirubinemia on or after postoperative day 5 [25]. Bile leakage was defined as the occurrence of drainage fluid containing at least 3-fold higher bilirubin concentration than the serum bilirubin concentration on or after postoperative day 3, or as the need for radiological or surgical intervention due to biliary collection or bile peritonitis [26]. Wound infection was defined as the detection of bacteria in the wound exudate.

### Follow-up

All patients were followed up at one month after surgery, then every 3 months for the rest of the first year, and every 6 months thereafter. Follow-up visits consisted of a physical examination, liver function tests, measurement of serum alpha-fetoprotein (AFP), abdominal ultrasonography and CT or magnetic resonance imaging (MRI). Postoperative antiviral therapy was rarely administered. OS was calculated as the time from the date of surgery until death from cancer, or complications due to underlying liver disease, or the date of the last follow up. DFS was calculated as the time from the date of surgery until detection of recurrent tumors or until the date of the last follow up without recurrence.

### Treatment of recurrence

All patients showing recurrence or metastasis were evaluated for new treatment. Patients with intrahepatic recurrence were managed with repeat hepatectomy, radiofrequency ablation (RFA), percutaneous ethanol injection (PEI), or transarterial chemoembolization (TACE), depending on the severity of hepatic dysfunction and tumor number, size, and location. Patients with resectable metastatic tumors were treated by metastasectomy.

### Propensity score matching

Propensity score analysis was used to reduce the bias in patient selection in observational studies. It seeks to eliminate confounding similarly to randomization, by creating comparison arms with similar distributions of measured baseline covariates [27]. Propensity scores were estimated using a logistic regression model based on age, gender, status of hepatitis B virus (HBV) and hepatitis C virus (HCV) infection, presence of diabetes mellitus, hypertension, cardiovascular diseases and respiratory diseases, platelet count, tumor number, tumor size, presence of liver cirrhosis, presence of tumor capsule, Barcelona Clinic Liver Cancer (BCLC) classification, AFP level, type of resection (minor or major), and liver function tests, including total bilirubin, alanine aminotransferase (ALT), aspartate aminotransferase (AST), albumin, and PT. Propensity score matching was performed using a 1:2 ratio without replacement and a caliper width of 0.1. The resulting subsets of score-matched obese and non-obese patients were used in subsequent analyses.

### Statistical analysis

Clinicopathological variables, mortality, morbility, and follow up data were summarized in S1 File. For continuous data, parametric analyses were performed using the *t* test; non-parametric analyses were performed using the Mann-Whitney *U* test. Categorical data were compared using the χ^2^ test or Fisher's exact test. OS and DFS were analyzed using the Kaplan-Meier method, and inter-group comparisons were performed using the log-rank test. Factors determined to be significant for OS and DFS using univariate analysis were introduced into a multivariate Cox proportional hazards model to determine adjusted hazard ratios (HRs) and associated 95% confidence intervals (CIs). All analyses were performed using SPSS 19.0 (IBM, USA). All statistical tests were two-sided, and *P* < 0.05 was considered statistically significant.

## Results

### Characteristics of all study patients

During the study period, 310 consecutive HCC patients with Child-Pugh A liver function undergoing curative hepatectomy were enrolled. Of these, 68 (22%) were obese based on BMI, and 242 (78%) were non-obese (Table 1). The obese group showed significantly evaluated levels of albumin and AST than did the non-obese group (*P* = 0.036, *P* = 0.043). However, the non-obese group showed a significantly higher frequency of multiple tumors (*P* = 0.006). The two groups were similar in age, gender, prevalence of diabetes mellitus, hypertension, cardiovascular diseases and respiratory diseases, HBV and HCV infection, serum bilirubin, AFP, ALT levels, platelet count, PT, tumor size, presence of tumor capsule, liver cirrhosis, BCLC stage and resection type (all *P* > 0.05). In addition, more than 89% of our cohort was infected with chronic HBV or HCV in both groups.

**Table 1:** Clinicopathological variables in non-obese patients (BMI <25 kg/m^2^) and obese patients (BMI ≥25 kg/m^2^) with hepatocellular carcinoma from southeast China who underwent curative resection.

Variable	Before Propensity Matching			After Propensity Matching		
	Non-obese	Obese	*P*	Non-obese	Obese	*P*
	(n = 242)	(n = 68)		(n = 115)	(n = 61)	
BMI, kg/m^2^	21.2±2.0	27.0±1.8	**<0.001**	21.2±2.6	26.8±1.8	**<0.001**
Age, yr	49.2±11.9	49.8±10.4	0.723	49.6±11	50.3±10.6	0.728
Male, n (%)	210 (86.8)	61 (89.7)	0.520	102 (88.7)	54 (88.5)	0.973
HBsAg positive, n (%)	209 (86.4)	54 (79.4)	0.158	96 (83.5)	50 (82.0)	0.800
HCV antibody-positive, n (%)	10 (4.1)	7 (10.3)	0.095	7 (6.1)	4 (6.6)	1.000
Comorbid disease, n (%)						
Diabetes mellitus	17 (7)	4 (5.9)	0.954	3 (2.6)	2 (3.3)	1.000
Hypertension	45(18.6)	18(26.5)	0.154	23(20)	14(23)	0.648
Cardiovascular diseases	27(11.2)	6(8.8)	0.581	17(14.8)	6(9.8)	0.354
Respiratory diseases	20(8.3)	7(10.3)	0.600	12(10.4)	5(8.2)	0.632
Total bilirubin level, μmol/L	14±6.5	13.9±6.5	0.968	14.4±6.7	14.0±6.5	0.671
AST, median (range), U/L	42.9 (24–53)	47.6 (30.3–55.8)	**0.043**	42.1 (25–49)	44.7 (29–55)	0.136
ALT, median (range), U/L	48.8 (29–54)	44.4 (31–55.8)	0.954	43.7 (29–50)	44.7 (31–55.5)	0.455
Albumin, g/L	40±4.6	41.3±4.6	**0.036**	41.5±4	41±4.3	0.446
Platelet count, 10^9^/L	178.9±75.9	177.6±73.1	0.900	174.2±63.4	176.0±73.9	0.865
Prothrombin time, s	12.9±1.6	12.9±1.9	0.765	12.9±1.5	12.9±1.9	0.999
AFP ≥400 ng/mL, n (%)	70 (28.9)	17 (25.0)	0.524	30 (26.1)	14 (23.0)	0.647
Solitary tumor, n (%)	165 (68.2)	58 (85.3)	**0.006**	93 (80.9)	51 (83.6)	0.654
Tumor size, median (range), cm	6 (3.5–8)	5.3 (3.6–6.6)	0.154	5.6 (3.5–7.5)	5.2 (3.5–6.6)	0.647
Tumor capsule, n (%)	135 (55.8)	42 (61.8)	0.379	49 (42.6)	23 (37.7)	0.529
Liver cirrhosis, n (%)	188 (77.7)	57(83.8)	0.272	95 (82.6)	51 (83.6)	0.867
BCLC stage A/B, n (%)	194 (80.2)/48 (19.8)	60(88.2)/8(11.8)	0.126	97 (84.3)/18 (15.7)	53 (86.9)/8 (13.1)	0.652
Minor/major resection, n (%)	105 (43.4)/137 (56.6)	31(45.6)/37(54.4)	0.747	56 (48.7) /59 (51.3)	34(55.7)/27(44.3)	0.374

Data are mean ± standard deviation or medium (25th-75th interquartile range) unless otherwise indicated

*Abbreviations*: AFP, alpha-fetoprotein; ALT, alanine aminotransferase; AST, aspartate aminotransferase; BCLC, Barcelona Clinic Liver Cancer.

### Mortality and morbidity

In the complete patient cohort, obese and non-obese groups showed similar 30-day mortality (1.5% vs. 2.5%, *P* = 1.000), 90-day mortality (4.4% vs. 5.0%, *P* = 0.706), and overall complication rate (25% vs. 17.4%, *P* = 0.156). Comparison of the distribution of postoperative complications between the two groups showed the only difference was the incidence of wound infection, which was significantly higher in the obese group than in the non-obese group (5.9% vs. 0.8%, *P* = 0.022; Table 2).

**Table 2:** Mortality and morbidity of non-obese and obese patients with hepatocellular carcinoma after curative resection.

Variable, n (%)	Before propensity matching			After propensity matching		
	Non-obese (n = 242)	Obese (n = 68)	*P*	Non-obese (n = 115)	Obese (n = 61)	***P***
30-day mortality	6 (2.5)	1 (1.5)	1.000	3(2.6)	1 (1.6)	1.000
90-day mortality	12(5.0)	2 (4.4)	0.706	5 (4.3)	2 (3.3)	1.000
*Postoperative complications*						
Any complication	42 (17.4)	17 (25)	0.156	19(16.5)	11 (18.0)	0.800
Pleural effusion	11 (4.5)	3 (4.4)	1.000	5 (4.3)	3 (4.9)	1.000
Pulmonary infection	7 (2.9)	2 (2.9)	1.000	4 (3.5)	2 (3.3)	1.000
Acute hepatic function failure	5 (2.1)	2 (2.9)	1.000	2 (1.7)	1 (1.6)	1.000
Postoperative abdominal bleeding	4 (1.7)	2 (2.9)	0.617	1 (0.9)	1 (1.6)	1.000
Bile leakage	4 (1.7)	0 (0)	0.580	1 (0.9)	0 (0)	1.000
Abdominal infection	3 (1.2)	1 (1.5)	1.000	2 (1.7)	0 (0)	0.544
Wound infection	2 (0.8)	4 (5.9)	**0.022**	2 (1.7)	2 (3.3)	0.610
Gastrointestinal hemorrhage	2 (0.8)	0 (0)	1.000	0 (0)	0 (0)	1.000
Delayed wound healing	2 (0.8)	2 (2.9)	0.210	1 (0.9)	2 (3.3)	0.276
Deep venous thrombosis	1 (0.4)	0 (0)	1.000	1 (0.9)	0 (0)	1.000
Liver abscess	1 (0.4)	0 (0)	1.000	0 (0)	0 (0)	1.000
Intestinal obstruction	0 (0)	1 (1.5)	0.219	0 (0)	0 (0)	1.000

In the propensity score-matched groups, the two groups showed similar 30-day mortality (1.6% vs. 2.6%, *P* = 1.000), 90-day mortality (3.3% vs. 4.3%, *P* = 1.000) and similar overall complication rates (18% vs. 16.5%, *P* = 0.800). Obese patients tended toward higher incidence of postoperative wound infection (3.3% vs. 1.7%, *P* = 0.610).

### Overall survival analysis of all study patients

**Fig 1:**
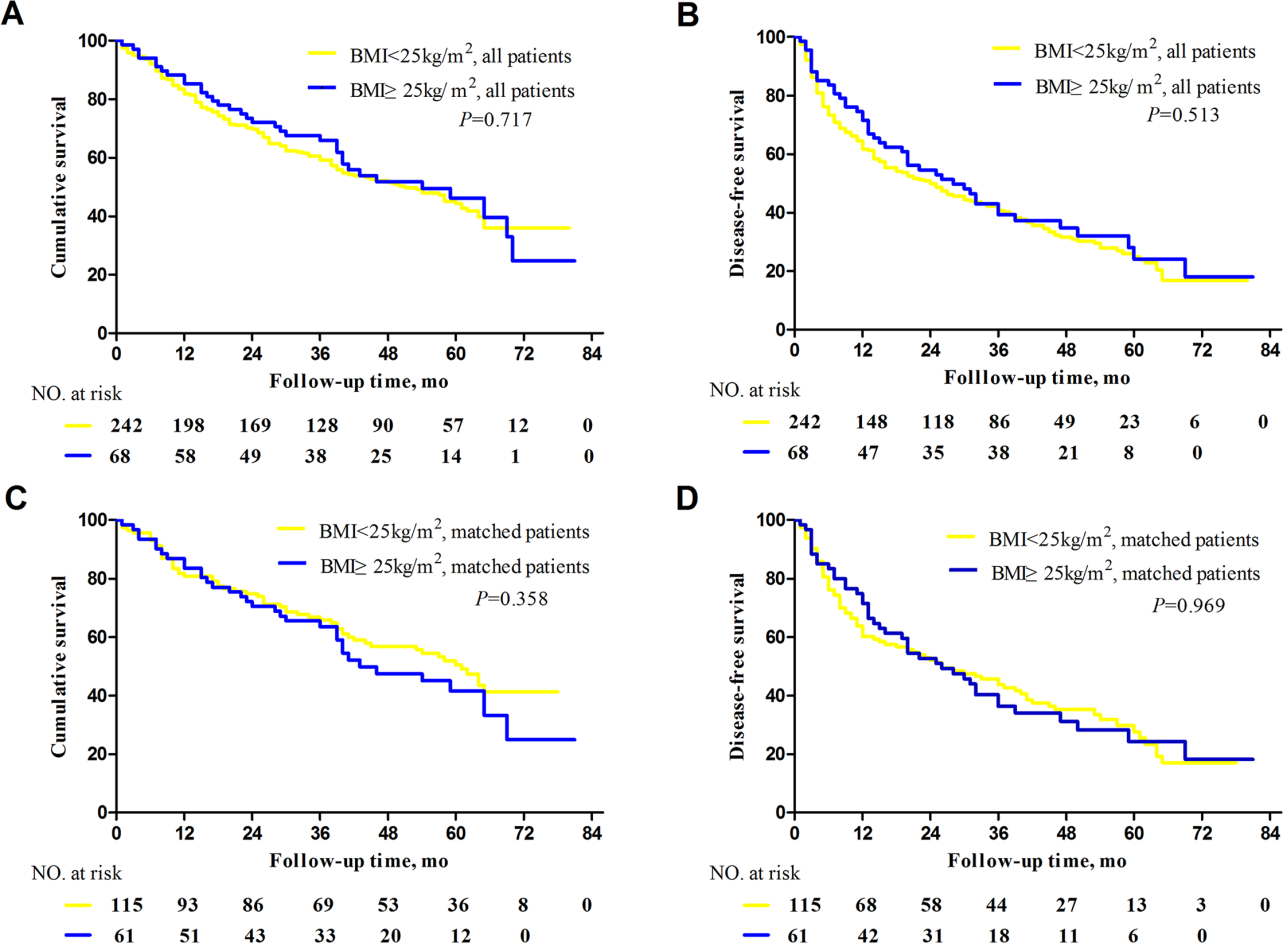
Survival of obese and non-obese patients with hepatocellular carcinoma after curative hepatectomy. Overall survival analyzed (A) before and (C) after propensity score matching. Disease-free survival (B) before and (D) after propensity score matching.

Median follow-up time was 38.7 months (range, 1–80 months) in the obese group and 38.1 months (range: 1–81 months) in the non-obese group. During follow-up, 35 patients (51%) in the obese group and 132 (55%) in the non-obese group died of cancer or complications due to underlying liver disease (*P* = 0.653). Estimated OS rates at 1, 3, and 5 years were 85.3%, 65.9%, and 46.2% in the obese group and 81.8%, 59.2%, and 44.4% in the non-obese group, respectively (*P* = 0.717, Fig 1A).

Dichotomized factors, including postoperative complications (present/absent), linked to survival were included in the survival analysis. Univariate analysis identified the following as prognostic factors predicting increased risk of mortality (Table 3): tumor size >5 cm (*P* < 0.001), BCLC B stage (*P* = 0.048) and major resection (*P* < 0.001). Of these factors, tumor size >5 cm was identified in multivariate Cox proportional hazard modeling as the only independent predictor of poor survival (HR 2.040, 95%CI 1.495 to 2.785, *P* < 0.001).

**Table 3:** Univariate and multivariate analysis of factors predictive of poor overall survival in the entire cohort of obese and non-obese HCC patients after curative resection.

Variables	Univariate analysis			Multivariate analysis		
	HR	95%CI	*P*	HR	95%CI	*P*
Tumor size (>5 cm)	2.040	1.495–2.785	<0.001	2.040	1.495–2.785	<0.001
BCLC stage (B)	1.458	1.004–2.119	0.046			
Major resection	1.973	1.430–2.722	<0.001			

*Abbreviations*: BCLC, Barcelona Clinic Liver Cancer; HR, hazard ration; 95%CI, 95% confidence interval.

### Disease-free survival analysis of all study patients

During follow-up, tumors recurred in 45 patients (66%) in the obese group and 173 (71%) in the non-obese group (*P* = 0.397). Estimated DFS rates at 1, 3, and 5years were 71.5%, 39.3%, and 32.1% in the obese group and 61.7%, 40.8%, and 25.0% in the non-obese group, respectively (*P* = 0.513, Fig 1B).

Univariate analysis identified the following factors as associated with higher incidence of tumor recurrence after curative hepatectomy: AFP level ≥400 ng/mL (*P* = 0.015), multiple tumors (*P* = 0.002), tumor size >5 cm (*P* < 0.001), BCLC B stage (*P* = 0.001), and major resection (*P* < 0.001). Multivariate analysis identified the following significant predictors of tumor recurrence (Table 4): tumor size >5 cm (HR 1.736, 95%CI 1.325 to 2.273, *P* < 0.001), and BCLC B stage (HR 1.650, 95%CI 1.188 to 2.290, *P* = 0.003).

**Table 4:** Univariate and multivariate analysis of factors predictive of poor disease-free survival in the entire cohort of obese and non-obese HCC patients after curative resection.

Variables	Univariate analysis			Multivariate analysis		
	HR	95%CI	*P*	HR	95%CI	*P*
AFP(≥400 ng/mL)	1.439	1.075–1.1927	0.015			
Tumor number (multiple)	1.583	1.188–2.109	0.002			
Tumor size (>5 cm)	1.784	1.363–2.334	<0.001	1.736	1.325–2.273	<0.001
BCLC stage (B)	1.740	1.254–2.413	0.001	1.650	1.188–2.290	0.003
Major resection	1.721	1.308–2.265	<0.001			

*Abbreviations*: AFP, alpha-fetoprotein; BCLC, Barcelona Clinic Liver Cancer; HR, hazard ration; 95%CI, 95% confidence interval.

### Propensity score matching of obese and non-obese patients

Propensity score matching in the ratio 1:2 led to selection of 61 obese patients and 115 non-obese patients. The matched groups showed similar baseline characteristics (Table 1), indicating that the matching procedure worked well.

### Overall survival analysis of propensity score-matched patients

Overall survival analysis of propensity-score matched patients showed that estimated OS at 1, 3, and 5 years was 83.6%, 63.6%, and 41.6% in the obese group, similar to the rates of 80.9%, 65.9%, and 49.1% in the non-obese group, respectively (*P* = 0.358, Fig 1C).

### Disease-free survival analysis of propensity score-matched patients

Disease-free survival analysis of propensity-score matched patients showed that estimated DFS at 1, 3, and 5 years was 71.5%, 36.3%, and 24.3% in the obese group, similar to the 60.2%, 43.7%, and 27.7% in the non-obese group, respectively (*P* = 0.969, Fig 1D).

### Subgroup analysis of all study patients

All HCC patients were categorized as non-obese (BMI <25 kg/m^2^), obese class I (BMI ≥25 kg/m^2^), or obese class II (BMI ≥30 kg/m^2^) groups. The non-obese, obese class I and obese class II groups comprised 242(78%), 61(20%) and 7(2%), respectively. The OS rates of the obese class I (85.2%, 65.2% and 47.6% at 1, 3, 5 years, repectively) and obese class II (85.7%, 71.4% and 35.7% at 1, 3, 5 years, repectively) groups did not significantly differ from the non-obese group (81.8%, 59.2% and 44.4% at 1, 3, 5 years, repectively; *P* = 0.669 vs. obese class I group and *P* = 0.792 vs. obese class II group). The DFS rates of the obese class I (71.5%, 40.9% and 23.1% at 1, 3, 5 years, repectively) and obese class II (71.4%, 28.6% and 28.6% at 1, 3, 5 years, repectively) groups also did not significantly differ from the non-obese group (61.7%, 40.8% and 23.1% at 1, 3, 5 years, repectively; *P* = 0.718 vs. obese class I group and *P* = 0.971 vs. obese class II group).

## Discussion

The present study suggests that obesity does not adversely affect OS, or risk of complications in HCC patients after curative resection. This is an important finding in light of the fact that obesity is directly associated with impairment of cardiac [28], pulmonary [29], and immunological functions [30], all of which can affect surgical outcomes.

The National Nutrition and Health Survey conducted in 2002 in China estimated that approximately 17.3% of Chinese are obese, based on the definition of BMI ≥25 kg/m^2^ [18]. In the present study, 68 of the 310 patients in our cohort (21.9%) were obese. This prevalence of obesity highlights the importance of understanding its effects on surgical risks and postoperative survival.

Studies of obesity and postoperative outcomes of various surgical procedures have given inconsistent results. Some authors have reported that elevated BMI is an independent predictor of morbidity [31,32], whereas others have argued that complications in obese patients are directly attributable to risk factors like smoking and diabetes, while high BMI by itself is not a risk factor for postoperative complications or death, with the possible exception of increased incidence of wound infection [33–36]. These differences may reflect the use of different definitions and classifications of obesity, different ways of reporting postoperative complications, differences in surgical procedure, and lack of statistical power. In the present study, the obese and non-obese groups showed similar mortality at 30 and 90 days and similar overall rates of postoperative complications following curative hepatectomy. Obese patients did show higher incidence of wound infection (5.9% vs. 0.8%, *P* = 0.022), consistent with the fact that obesity is a well-known risk factor for surgical site infection, especially wound infection, after hepatic resection [13,37]. However, this difference between our obese and non-obese cohorts became insignificant after adjusting for all confounding factors using propensity-score matching (3.3% vs. 1.7%, *P* = 0.610). It may be possible to counteract any added risk of infection in obese HCC patients using appropriate measures, such as a plastic adhesive drape impregnated with iodophor, a subcutaneous dressing impregnated with saline solution, and perioperative control of blood sugar level [8].

We measured similar OS for obese and non-obese patients, and obesity was not a risk factor for mortality in our cohort, as reported in other retrospective studies in Japan, Europe and America [11–17]. In fact, a few studies have suggested that elevated BMI is associated with improved OS [38,39]. This may reflect the fact that obese people have good nutritional and physiological reserves and show enhanced inflammatory response to injury, which may counteract comorbidity and mortality among obese patients undergoing hepatectomy [11,38]. On the other hand, some authors have reported that obesity worsens the prognosis of HCC patients [9,10]. Further studies are needed to clarify these differences before definitive conclusions can be made.

As with OS, we did not find significant differences in DFS between propensity score-matched obese and non-obese patients. This is consistent with several studies on obesity in HCC patients undergoing curative therapy [11,16,17]. HCC recurrence after curative hepatectomy occurs as a result of intrahepatic metastasis and multicentric carcinogenesis [40]. Theoretically, the former depends on factors related to HCC development such as tumor grade, vascular invasion, and microsatellite lesions; the latter, in contrast, is more likely related to underlying liver conditions such as cirrhosis and hepatitis. Obesity may enhance the risk of multicentric carcinogenesis by increasing oxidative stress, and it may hinder the detection of intrahepatic metastatic lesions, yet we found similar recurrence rates in obese and non-obese patients. It may be that obesity by itself does increase the risk of recurrence, but its effect is not as strong as those of other recurrence risk factors in Table 4. Further studies on primary HCC development are required to investigate the effects of obesity on hepatocarcinogenesis.

Our retrospective study is subject to several limitations. First, the sample size is relatively small, especially after propensity score matching. It would have been better to include a sufficiently large sample so that we could have analyzed patients in different BMI ranges, rather than somewhat arbitrarily adopting a single cut-off for obese or non-obese. Second, more than 89% of our cohort was infected with chronic HBV or HCV, unlike most HCC populations in other countries, the information of the severity of chronic hepatitis was unclear, and incidence of non-alcoholic steatohepatitis was also unclear in patients whose hepatitis was unrelated to either virus. Both these cohort characteristics may affect the accuracy of OS estimates. Third, BMI at diagnosis was used to classify patients as obese or non-obese, but weight may have changed substantially during follow-up; unfortunately, no data were collected on this. Lastly, follow-up was relatively short in this study. Our results should be confirmed in larger prospective studies with longer observation periods.

## Conclusions

Obesity does not significantly increase incidence of complications or reduce OS or DFS in HCC patients from southwest China following curative hepatectomy.

## Supporting Information

S1 File Clinicopathological variables, mortality, morbility, and follow up data of non-obese and obese patients with hepatocellular carcinoma after curative resection. (ZIP)
